# Supplementary material for: A Targeted Metabolomics Approach to Study Secondary Metabolites and Antioxidant Activity in ‘Kinnow Mandarin’ during Advanced Fruit Maturity
Source: Foods. 2022 May 13;11(10):1410. doi: 10.3390/foods11101410 (PMC9141733; doi:10.3390/foods11101410)
Supplement: Supplementary file 1 [file foods-11-01410-s001.zip › foods-1667397-supplementary.pdf]

Table S1. Agro–climatic conditions of two major Kinnow growing climates.

Sampling location	Agro–climatic zone	Latitude and longitude	Altitude (m)	Temperature Range (°C)	Average Annual Rainfall (mm)	Soil type
Abohar	Subtropical arid	30.15°N; 74.19°E	187	0–42	210	Sandy loam
Chaunni Kalan	Subtropical humid	31.5°N; 75.94°E	301	0–41	600	Sandy loam and loamy sand

Trewartha Climate classification has been used to define agro–climatic conditions of growing locations. Subtropical climates (located between 23.5 and 40° latitude) are characterised by warm to hot summers and cool winters with infrequent frost. The subtropical arid (STA) region has a dry climate where the rate of evaporation is more than the moisture received from precipitation (200–400 mm). Generally, the mean maximum and mean minimum temperatures are 46° C and 6° to 10° C, respectively.

In subtropical humid (STH) region, the climate is hot, usually humid summers and mild to cool winters. In most locations, the mean temperature of the coldest month is between 3 °C and 18 °C. The warmest month normally has a mean temperature of 22°C or higher. Rainfall (625 to 2500 mm) often shows a summer peak and monsoons are well developed.

Table S2. MRM parameters of polyphenolics and limonoids used for data acquisition in LC–MS/MS

S.No.	Compound Name	Chemical formula	Molecular mass	ESI	RT	Q1 (m/z)	Q3 (m/z)	DP (V)	CE (V)	CXP (V)
1.	Hesperidin	$C_{28}H_{34}O_{15}$	610.56	+	10.89	611	303	70	30	16
							449	70	15	25
2.	Narirutin	$C_{27}H_{32}O_{14}$	580.55	+	10.46	581	419	130	13	21
							273	130	29	14
3.	Naringin	$C_{27}H_{32}O_{14}$	580.53	+	10.57	581	273	160	16	14
							419	160	12	24
4.	Naringenin	$C_{15}H_{12}O_5$	272.25	+	10.57	273	153	145	29	18
							147	145	14	16
5.	Neoeriocitrin	$C_{27}H_{32}O_{15}$	596.53	+	12.06	597	289	150	23	15
							435	150	14	24
6.	Kaempferol	$C_{15}H_{10}O_6$	286.24	+	12.06	287	153	80	45	12
							121	80	47	13
7.	Daidzein	$C_{15}H_{10}O_4$	254.24	+	13.55	255	181	160	38	8
							199	160	33	10
							137	160	35	15
							91	160	43	10
8.	Quercetin	$C_{15}H_{10}O_7$	302.24	+	10.89	303	229	145	38	11

							153	145	42	18
9.	Rutin	C27H30O16	610.52	+	10.89	611	303	110	25	15
							465	110	17	24
10.	Quercetin−3−O−galactoside	C21H20O12	464.38	+	10.78	465	303	120	16	15
11.	Tangeretin	C20H20O7	372.37	+	17.08	373	343	208	32	17
12.	Nobiletin	C21H22O8	402.39	+	16.12	403	373	260	36	31
							388	260	18	20
13.	Quercetin−3−O−glucoside	C21H20O12	264.38	+	10.79	465	303	170	16	15
14.	Myricetin	C15H10O8	318.24	−	9.34	317	179	−80	−27	−18
							151	−80	−29	−14
							137	−80	−30	−15
15.	Taxifolin	C15H12O7	304.25	+	10.81	305	259	135	19	13
							153	135	20	14
16.	Sinapic Acid	C11H12O5	224.21	+	9.24	225	207	123	13	10
							175	123	19	18
17.	Ellagic Acid	C14H6O8	302.2	−	10.84	301	285	−123	−43	−30
							144	−123	−49	−19
18.	Ferulic Acid	C10H10O4	184.18	+	9.29	195	177	80	14	23
							145	80	21	16

							125	90	15	13
19.	Vanillin	C8H8O3	152	+	10.94	153	93	90	20	10
							65	90	30	9
20.	Benzoic Acid	C7H6O2	162.16	−	9.4	121	77	−60	−16	−12
21.	Caffeic Acid	C9H8O4	180.18	+	9.58	181	163	107	13	13
							145	107	21	13
22.	p−coumaric Acid	C9H8O3	184.18	+	3.41	165	147	90	15	19
							119	90	24	14
							155	101	11	15
23.	Syringic Acid	C9H10O5	188.17	+	2.99	199	140	101	20	13
							123	101	16	21
24.	t−cinnamic acid	C9H8O2	142.18	−	7.05	147	103	−60	−18	−15
25.	Luteolin	C15H10O6	286.24	+	11.99	287	153	90	40	18
							135	90	43	15
26.	Apigenin	C15H10O5	270.24	+	10.42	271	153	70	40	17
							119	70	38	19
27.	Epigallocatechin	C15H14O7	306.27	−	10.58	305	125	−150	−25	−17
							179	−150	−21	−10
28.	Catechin	C15H14O6	290.27	−	9.59	289	203	−130	−28	−16
							123	−130	−38	−12
29.	Epicatechin Gallate	C22H18O10	442.37	−	9.48	441	289	−125	−21	−12

							161	−125	−22	−10
30.	Phloridzin dihydrate	C2H24O10.<br>H2O	472.44	−	11.11	435	273	−106	−23	−26
31.	Genistein	C15H10O5	270.24	+	10.47	271	153	150	35	18
							215	150	37	26
32.	Limonin	C26H30O8	470.51	+	16.13	471	425	180	26	22
							161	180	30	10
33.	Nomilin	C28H34O9	514.56	+	16.13	515	456	180	22	20
							161	170	32	22
34.	Obacunone	C26H30O7	454.51	+	15.01	455	391	170	22	20
							427	180	26	22
35.	Limonin glucoside	C32H42O14	650.67	−	6.35	649	605	−185	−36	−9
							443	−185	−40	−24

Table S3. Limits of detection and quantification (LOD, LOQ), calibration ranges, and appropriate correlation coefficients r of all the quantified secondary metabolites.

Metabolites	LOQ (μg/L)	LOD (μg/L)	Correlation coefficient (r)	Calibration range (μg/L)
Hesperidin	61.87	187.50	0.9980	100−1000
Naringenin	197.20	597.58	0.9986	200−3000
Naringin	23.82	72.17	0.9980	100−800
Narirutin	33.44	101.34	0.9981	100−2000
Neoeriocitrin	17.71	53.67	0.9994	50−800
Quercetin−3−o−glucoside	0.40	1.21	0.9991	1−30
Kaempferol	20.86	63.21	0.9989	50−500
Myricetin	0.93	2.82	0.9999	1−20
Nobiletin	5.95	18.02	0.9976	10−300
P−coumaric acid	7.64	23.14	0.9993	10−100
Quercetin−3−o−glactoside	94.49	286.33	0.9964	100−2000
Quercetin	19.51	59.12	0.9993	20−500
Rutin	19.76	59.89	0.9999	20−1000
Tangeretin	22.69	68.77	0.9996	50−500
Taxifolin	4.98	15.11	0.9977	5−50
Benzoic acid	0.28	0.84	0.9994	30−300
Caffeic acid	2.46	7.47	0.9993	5−50
Chlorogenic acid	3.30	9.99	0.9983	5−50
Ellagic acid	14.64	44.36	0.9996	20−200
Ferulic acid	129.33	391.91	0.9976	300−3000
Sinapic acid	18.94	57.40	0.9956	20−500
Syringic acid	0.71	2.14	0.9987	1−20
vanillin	1.94	5.87	0.9987	2−30
Catechin	0.85	2.57	0.9984	1−10
Epicatechin gallate	0.25	0.77	0.9998	1−20
Epigallocatechin	19.88	60.23	0.9991	30−800

Apigenin	4.88	14.80	0.9999	5−200
Luteolin	22.60	68.48	0.9995	50−500
Daidzein	1.61	4.87	0.9982	2−50
Genstein	1.69	5.12	0.9999	2−100
Phloridzin	2.69	8.14	0.9984	3−50
Limonin	220.86	669.26	0.9994	200−5000
Nomilin	7.48	22.67	0.9986	10−300
Obacunone	1.28	3.87	0.9989	2−20
Limonin glucoside	2149.59	6513.91	0.9994	2.5−20

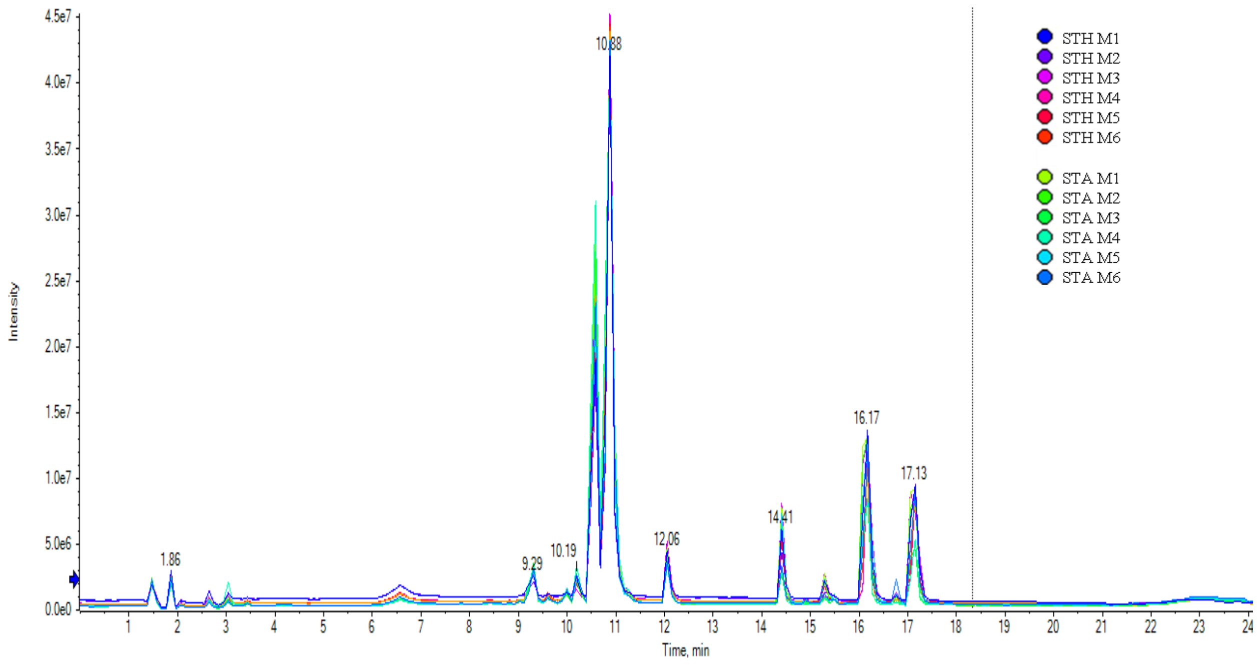

Figure S1. Total ion chromatogram (TIC) of phenolic compounds in subtropical−humid (STH) and subtropical−arid (STA) at six different maturity stages (M1 to M6).

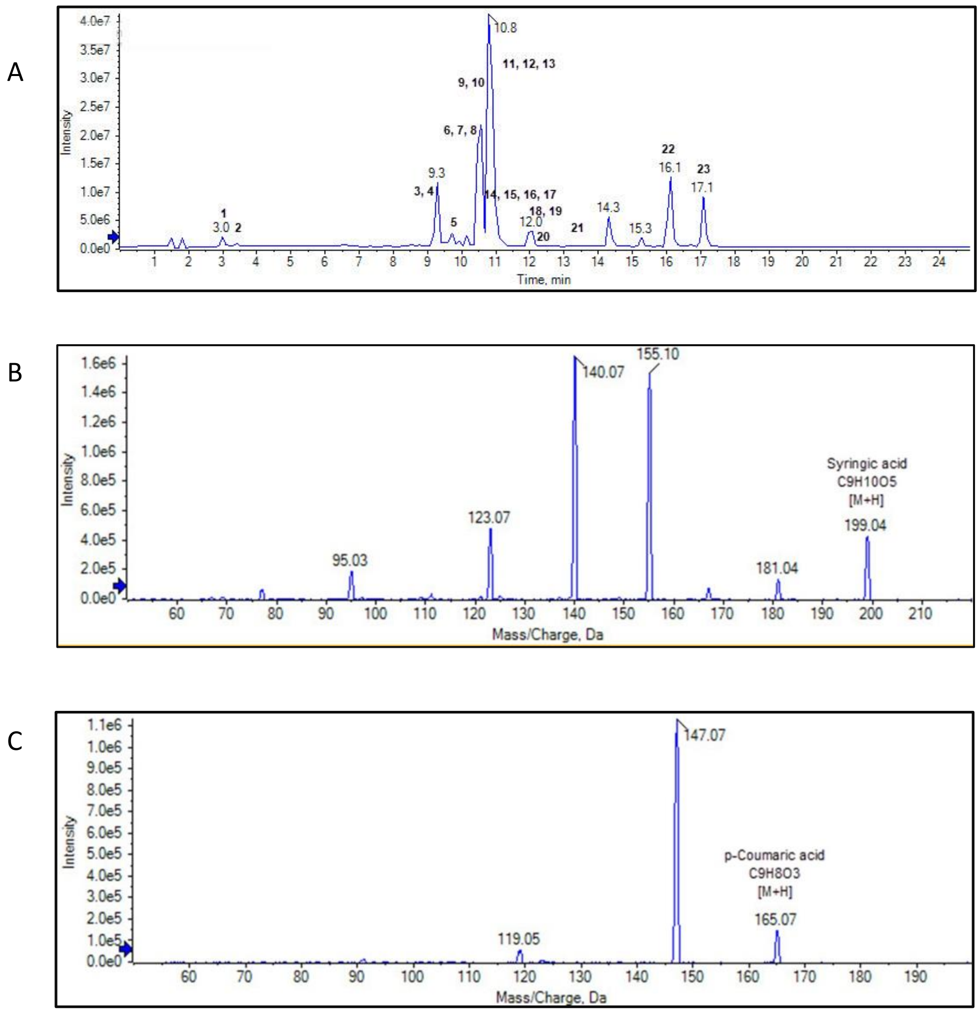

D

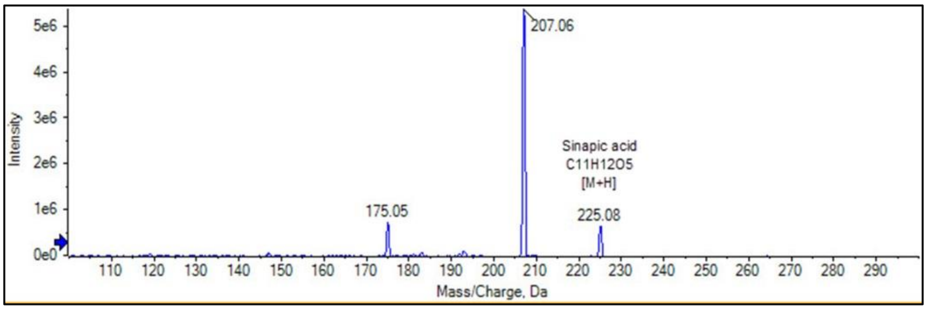
207.06
Sinapic acid
C11H12O5
[M+H]
175.05
225.08
Intensity
Mass/Charge, Da

E

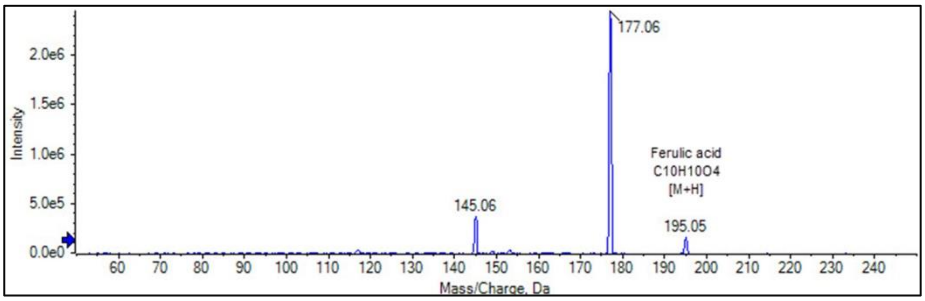
177.06
Ferulic acid
C10H10O4
[M+H]
145.06
195.05
Intensity
Mass/Charge, Da

F

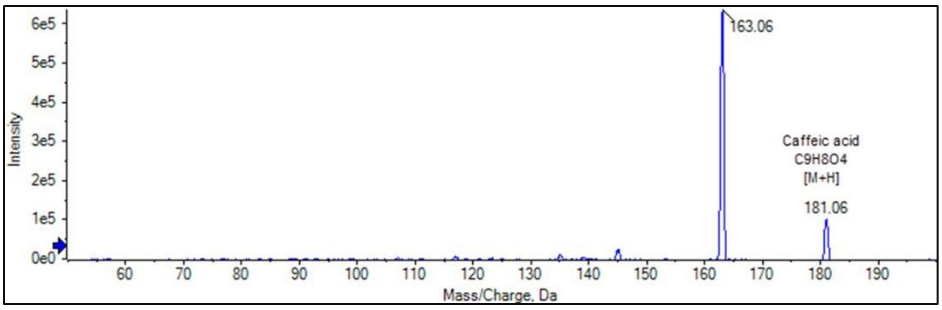
163.06
Caffeic acid
C9H8O4
[M+H]
181.06
Intensity
Mass/Charge, Da

G

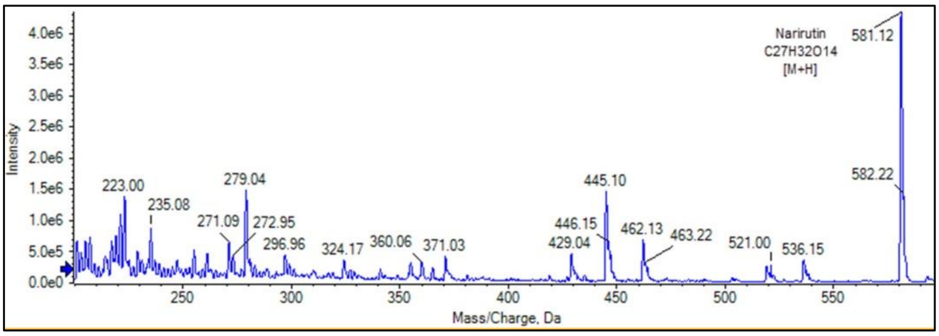
Narirutin
C27H32O14
[M+H]
581.12
582.22
223.00
279.04
235.08
271.09
272.95
296.96
324.17
360.06
371.03
445.10
446.15
429.04
462.13
463.22
521.00
536.15
Intensity
Mass/Charge, Da

H

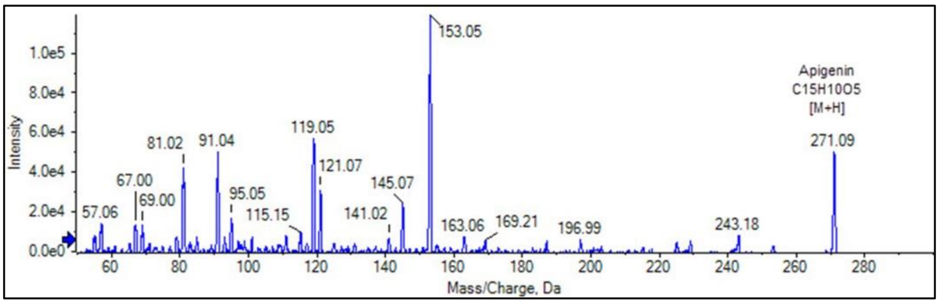
153.05
Apigenin
C15H10O5
[M+H]
271.09
119.05
81.02
91.04
121.07
67.00
145.07
95.05
69.00
57.06
115.15
141.02
163.06
169.21
196.99
243.18
Intensity
Mass/Charge, Da

I

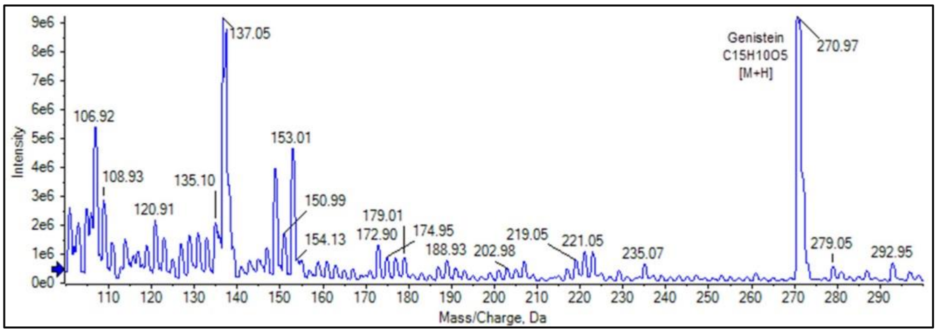
Genistein
C15H10O5
[M+H]
270.97
137.05
106.92
108.93
135.10
120.91
153.01
150.99
154.13
179.01
172.90
174.95
188.93
202.98
219.05
221.05
235.07
279.05
292.95
Intensity
Mass/Charge, Da

J

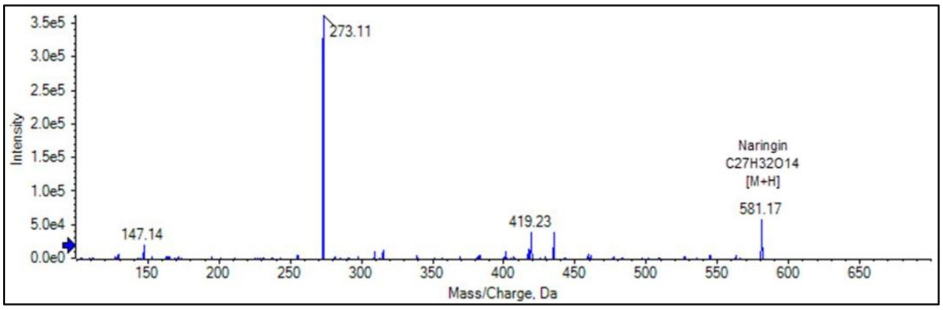
273.11
Naringin
C27H32O14
[M+H]
581.17
419.23
147.14
Intensity
Mass/Charge, Da

K

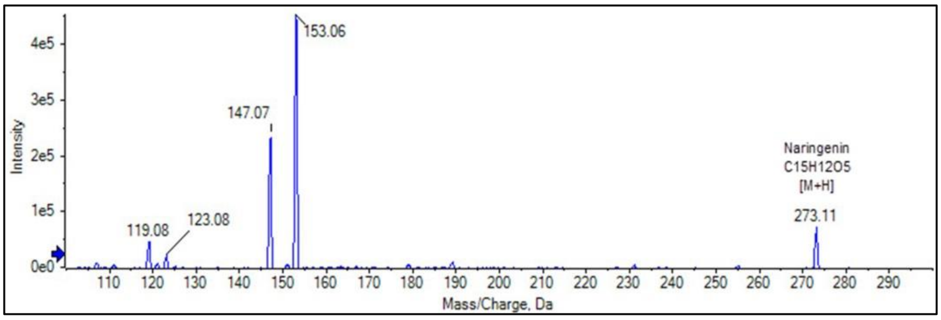
153.06
147.07
Naringenin
C15H12O5
[M+H]
273.11
119.08
123.08
Intensity
Mass/Charge, Da

L

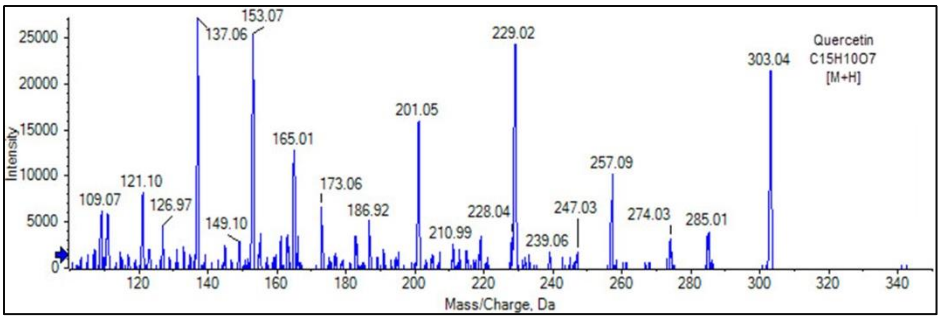
137.06
153.07
229.02
303.04
Quercetin
C15H10O7
[M+H]
201.05
165.01
257.09
121.10
109.07
126.97
173.06
186.92
149.10
228.04
247.03
274.03
285.01
210.99
239.06
Intensity
Mass/Charge, Da

M

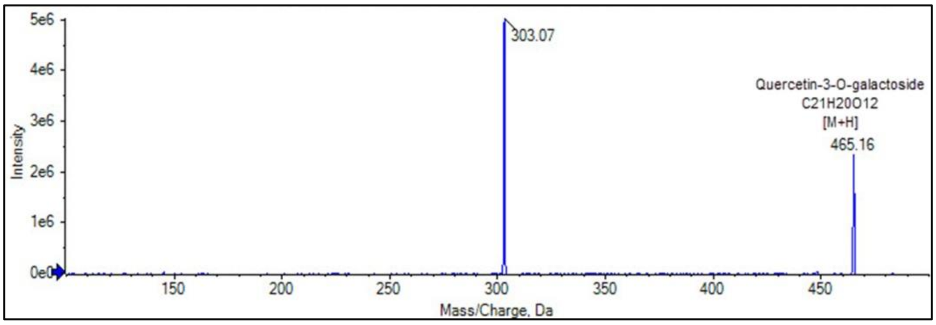
303.07
Quercetin-3-O-galactoside
C21H20O12
[M+H]
465.16
Intensity
Mass/Charge, Da

N

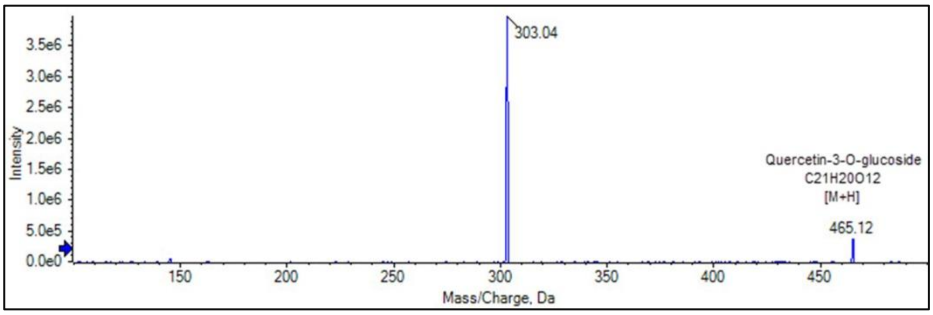
303.04
Quercetin-3-O-glucoside
C21H20O12
[M+H]
465.12
Intensity
Mass/Charge, Da

O

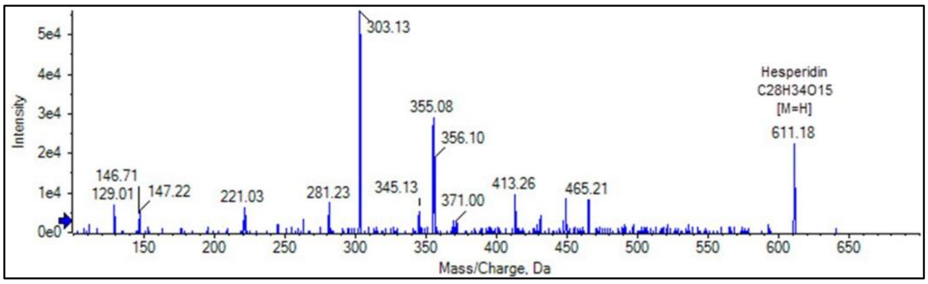
303.13
Hesperidin
C28H34O15
[M=H]
355.08
356.10
611.18
146.71
129.01
147.22
221.03
281.23
345.13
371.00
413.26
465.21
Intensity
Mass/Charge, Da

P

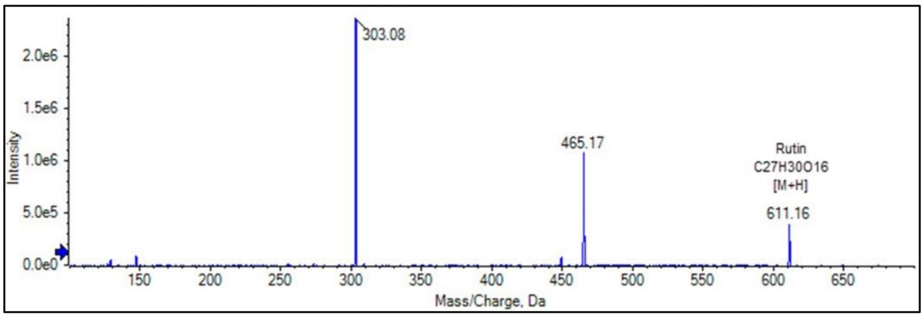
303.08
465.17
Rutin
C27H30O16
[M+H]
611.16
Intensity
Mass/Charge, Da

Q

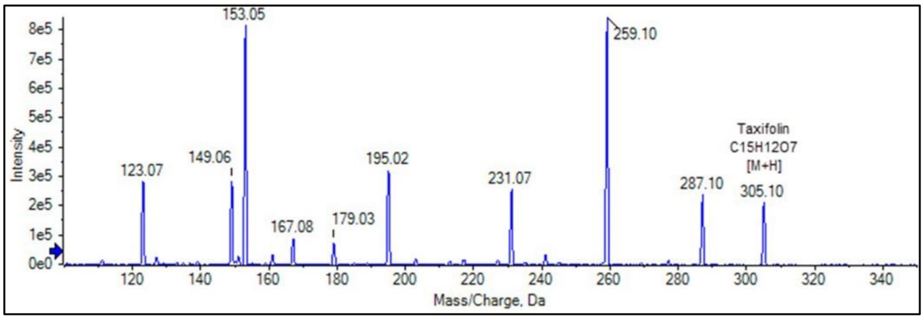
153.05
259.10
Taxifolin
C15H12O7
[M+H]
123.07
149.06
195.02
231.07
287.10
305.10
167.08
179.03
Intensity
Mass/Charge, Da

R

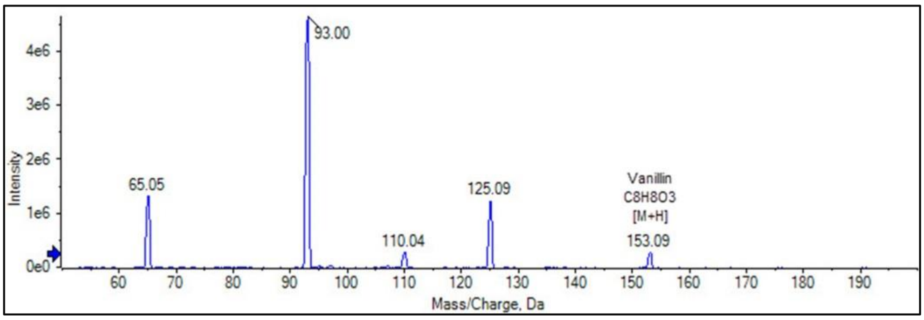
93.00
65.05
125.09
Vanillin
C8H8O3
[M+H]
110.04
153.09
Intensity
Mass/Charge, Da

S

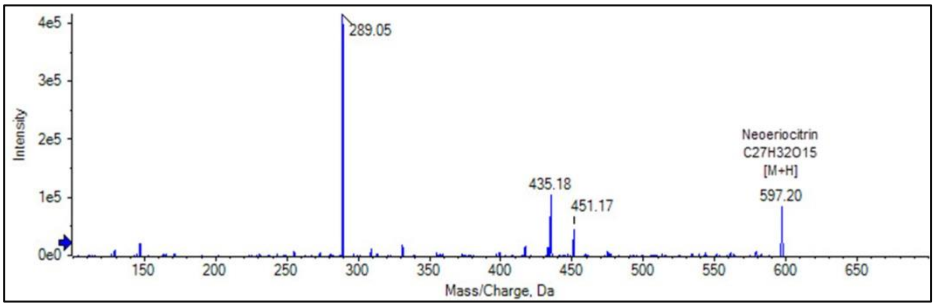
Neoeriocitrin
C27H32O15
[M+H]
597.20
289.05
435.18
451.17
Intensity
Mass/Charge, Da

T

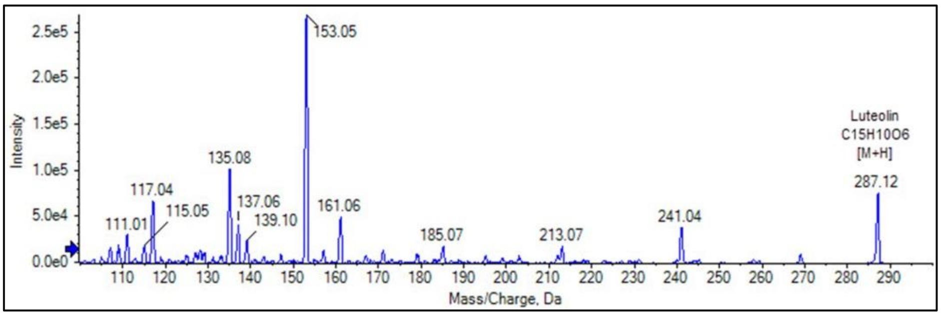
Luteolin
C15H10O6
[M+H]
287.12
153.05
135.08
117.04
111.01
115.05
137.06
139.10
161.06
185.07
213.07
241.04
Intensity
Mass/Charge, Da

U

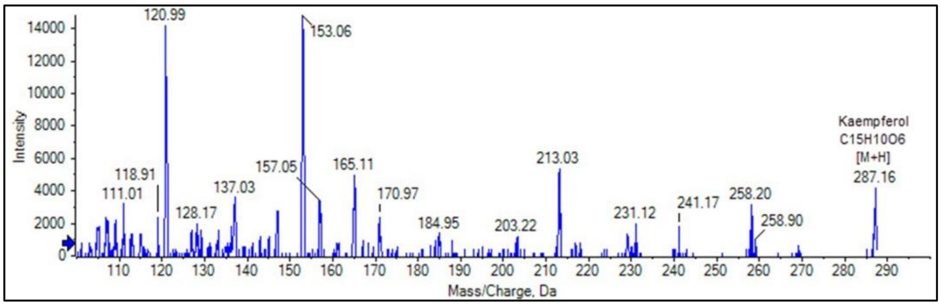
Kaempferol
C15H10O6
[M+H]
287.16
120.99
153.06
118.91
111.01
128.17
137.03
157.05
165.11
170.97
184.95
203.22
213.03
231.12
241.17
258.20
258.90
Intensity
Mass/Charge, Da

V

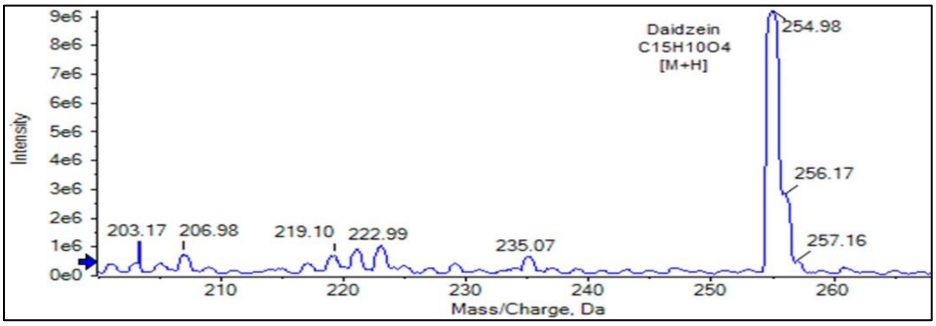
Daidzein
C15H10O4
[M+H]
254.98
256.17
257.16
203.17
206.98
219.10
222.99
235.07
Intensity
Mass/Charge, Da

W

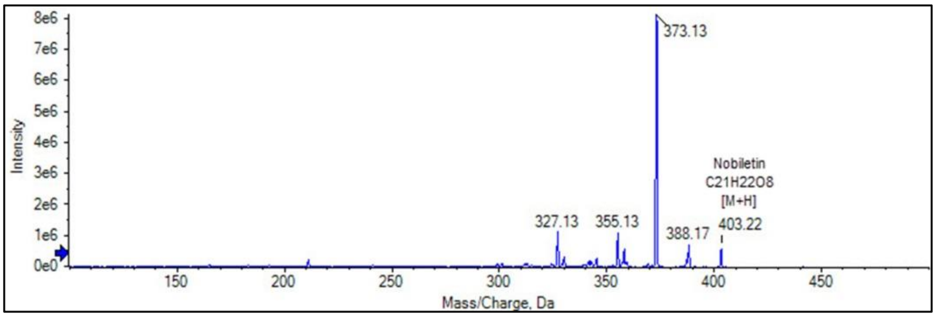
Nobiletin
C21H22O8
[M+H]
403.22
373.13
327.13
355.13
388.17
Intensity
Mass/Charge, Da

X

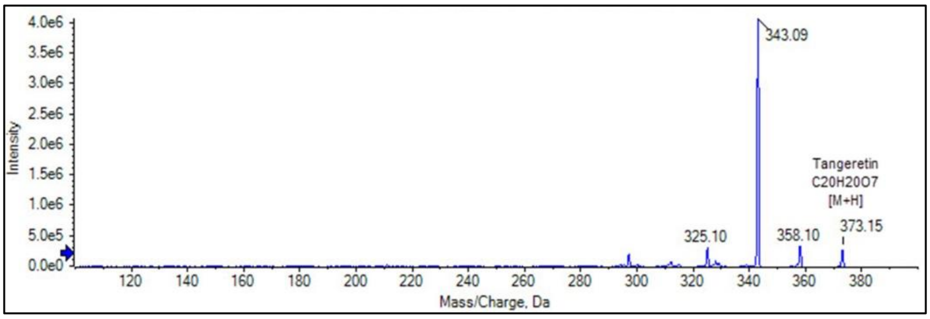

Figure S2. (A) Total ion chromatogram and mass spectras of polyphenolics analysed in positive ionization mode; B) syringic acid (m/z 199.04), C) p−coumaric acid (m/z 165.07), D) sinapic acid (m/z 225.08), E) ferulic acid (m/z 195.05), F) caffeic acid (m/z 181.06), G) narirutin (m/z 581.12), H) apigenin (m/z 271.09), I) genistein (m/z 270.97), J) naringin (m/z 581.17), K) naringenin (m/z 273.11), L) quercetin (m/z 303.04), M) quercetin−3−O−galactoside (m/z 465.16), N) quercetin−3−O−glucoside (m/z 465.16), O) hesperidin (m/z 611.18), P) rutin (m/z 611.16), Q) taxifolin (m/z 305.10), R) vanillin (m/z 153.09), S) neoeriocitrin (m/z 597.20), T) luteolin (m/z 287.12), U) kaempferol (m/z 287.16), V) daidzein (m/z 256.17), W) nobiletin (m/z 403.22), X) tangeretin (m/z 373.15).

A

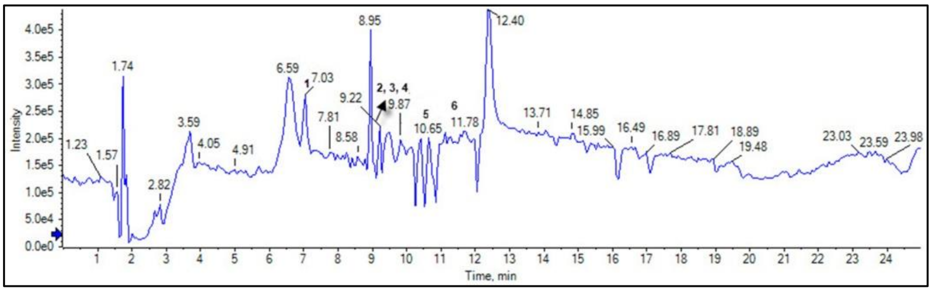

B

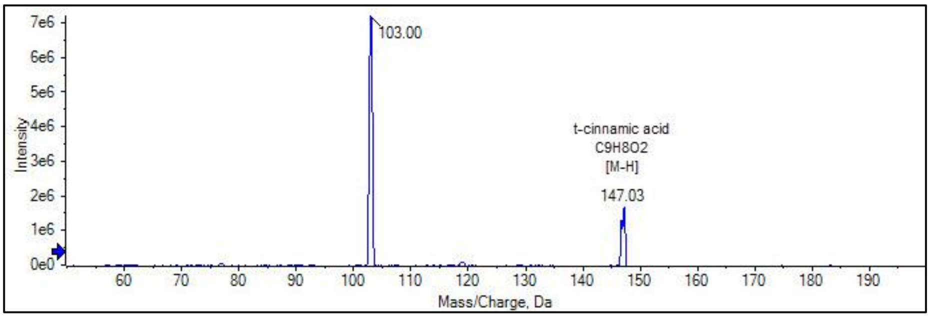

C

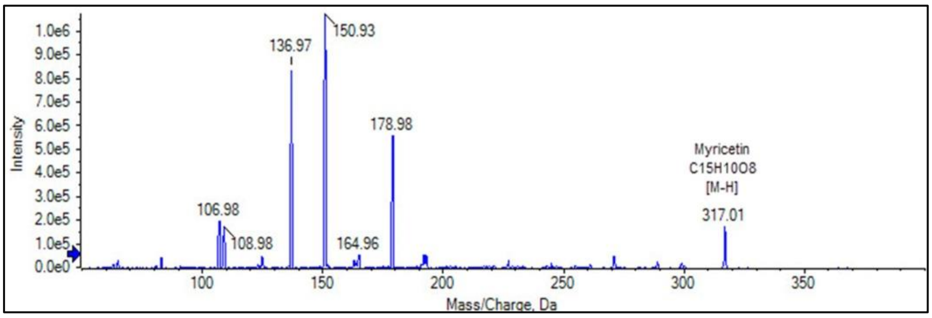

D

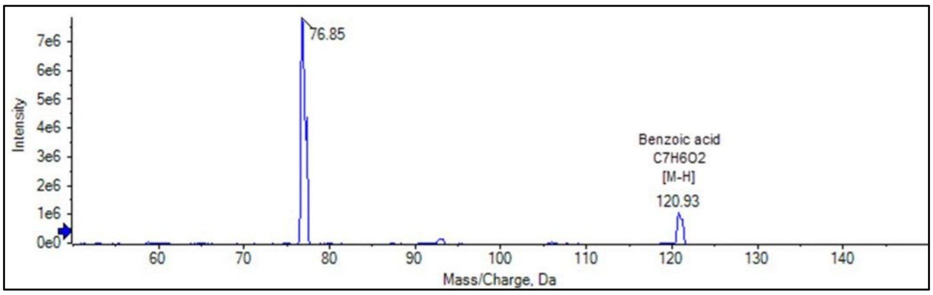

E

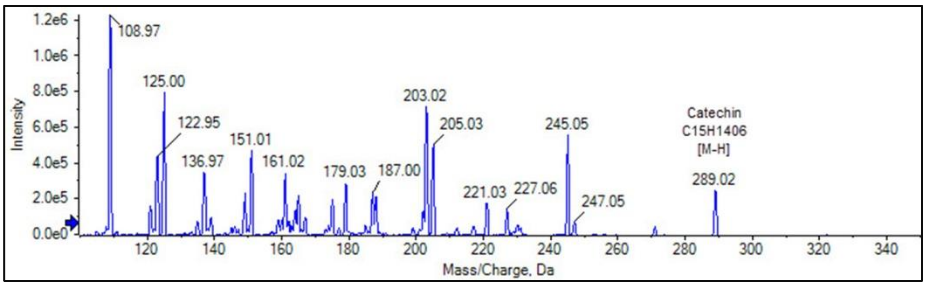

F

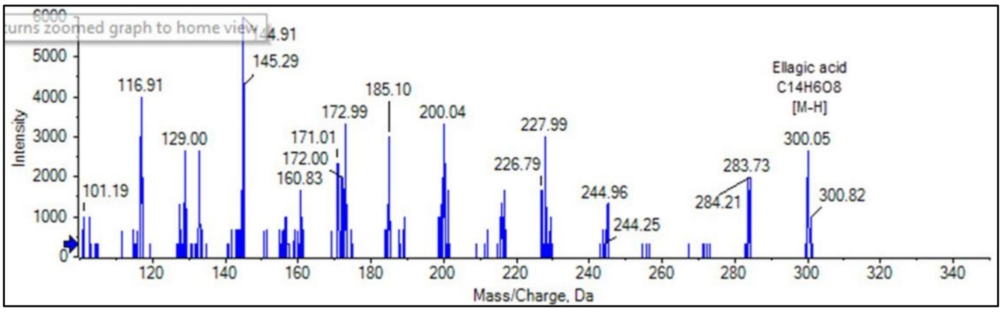

G

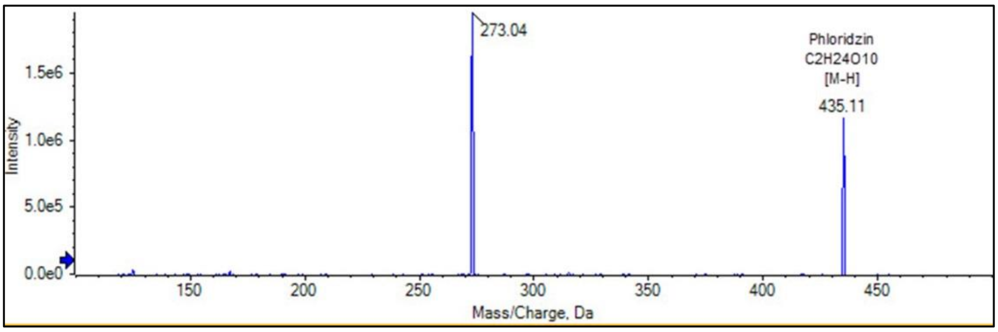

Figure S3. (A) Total ion chromatogram and mass spectra of polyphenolics analysed in negative ionization mode; B) t−cinnamic acid (m/z 147.03), C) myricetin (m/z 317.01), D) benzoic acid (m/z 120.93), E) catechin (m/z 289.02), F) ellagic acid (m/z 300.82), G) phlordzin (m/z 435.11).

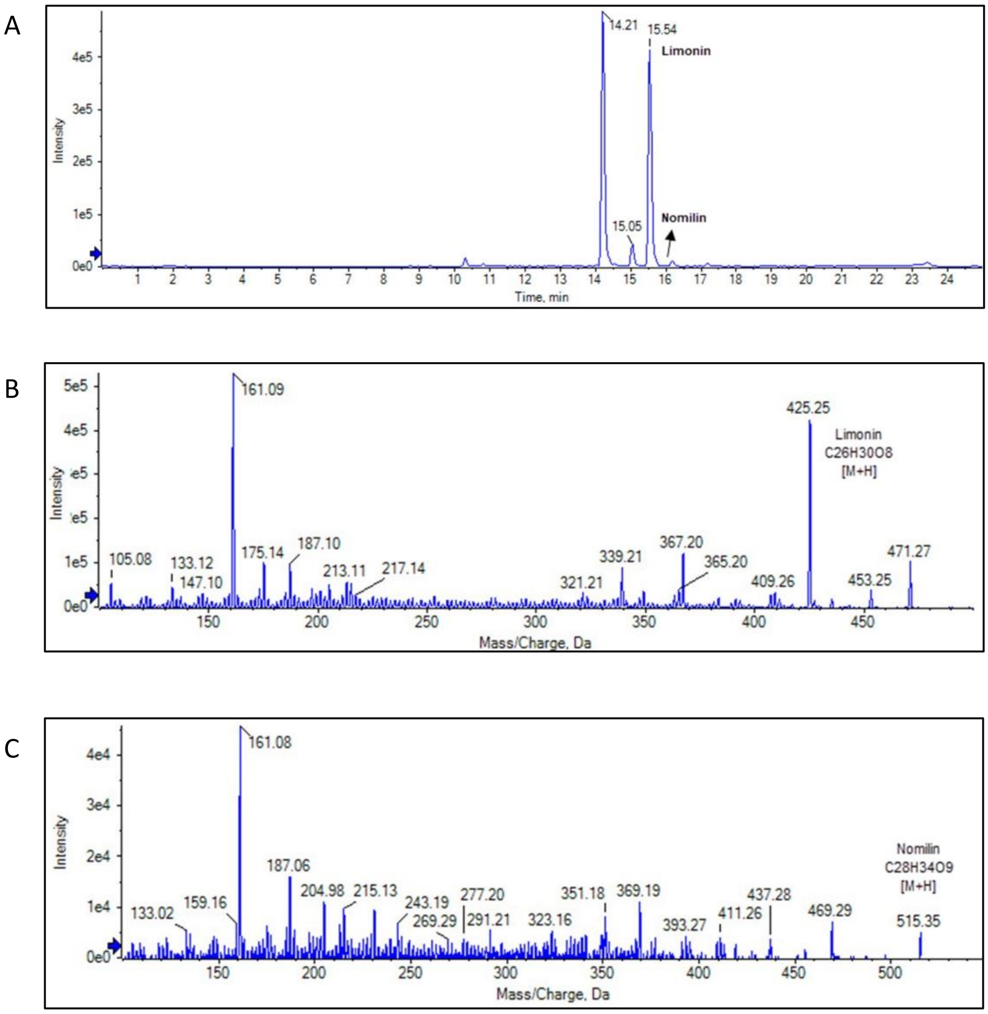

Figure S4. (A) Total ion chromatogram and mass spectra of limonoid aglycones analysed in positive ionization mode; B) limonin (m/z 471.27), C) nomilin (m/z 515.35), D).

A

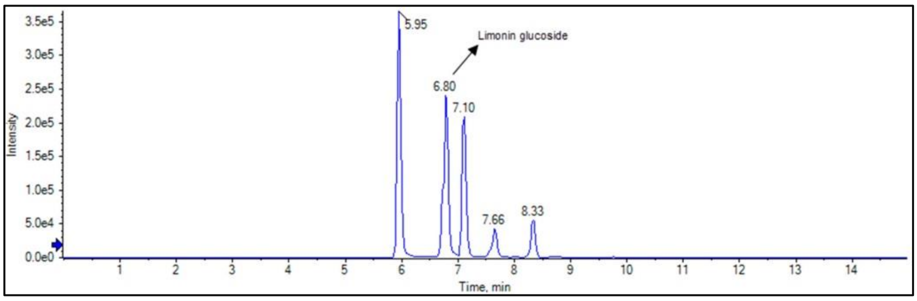

B

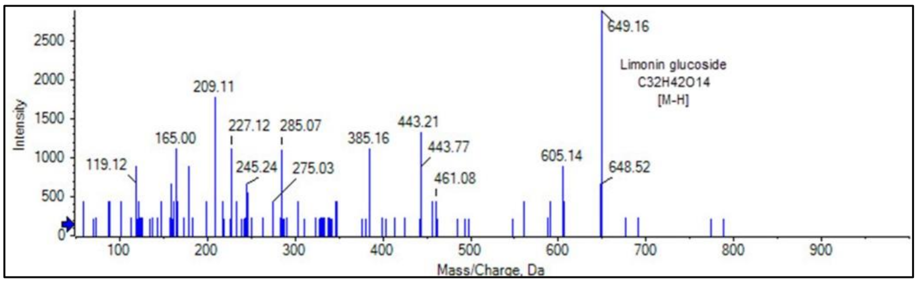

Figure S5. (A) Total ion chromatogram and mass spectra of limonoid glucosides analysed in positive ionization mode; B) limonin glucoside (m/z 649.16)
